# The Clinical Value of Autoantibodies in Rheumatoid Arthritis

**DOI:** 10.3389/fmed.2018.00339

**Published:** 2018-12-03

**Authors:** Serena Bugatti, Antonio Manzo, Carlomaurizio Montecucco, Roberto Caporali

**Affiliations:** Division of Rheumatology and Early Arthritis Clinic, IRCCS Policlinico San Matteo Foundation, University of Pavia, Pavia, Italy

**Keywords:** rheumatoid arthritis, autoantibodies, rheumatoid factor, anti-citrullinated protein antibodies, remission

## Abstract

Rheumatoid arthritis (RA) is a highly heterogeneous syndrome in terms of clinical presentation, progression, and response to therapy. In such a complicated context, the identification of disease-related biomarkers would be undoubtedly helpful in assisting tailored approaches for every patient. Despite remarkable efforts, however, progress in new biomarker development and validation is dramatically slow. At present, none of the candidate genetic, cellular, or molecular biomarker has yet surpassed the clinical value of RA-specific autoantibodies, including rheumatoid factor (RF) and anti-citrullinated protein autoantibodies (ACPA). Rather, recent years have witnessed significant advancements in our understanding of the multiple roles that RF and ACPA play in RA pathophysiology. This has helped clarifying the mechanistic basis of the clinical associations of autoantibodies in RA. In this short review, we will briefly summarize the effector functions of RF and ACPA, and analyse how autoantibodies may help subclassifying RA patients in terms of clinical presentation and response to therapy.

Our knowledge on rheumatoid arthritis (RA) has dramatically increased over the past 2 decades. Recognition that RA starts early and key processes of damage even develop before clinically apparent arthritis have fuelled the processes of early diagnosis and prompt treatment institution within the window of opportunity (1, 2). In parallel, better understanding of disease pathogenesis has helped developing new targeted therapeutics that allow good control of signs and symptoms and improve the overall outcomes of the disease (3). Despite these remarkable advances, however, the management of patients with RA remains imprecise. It is widely accepted that RA encompasses great heterogeneity in clinical presentation and outcomes. Yet, any attempt of personalizing treatment based on patients' genotypic and phenotypic characteristics has been largely unsuccessful (4, 5). Despite considerable efforts in producing new taxonomies, the most robust distinction of RA subtypes remains the classification based on the autoantibody status. Patients with rheumatoid factor (RF) and anti-citrullinated protein antibodies (ACPA) have specific genetic and environmental risk factors, and differ from autoantibody-negatives in their clinical course and prognosis (6). The recent discovery of the direct pathogenetic roles of autoantibodies in RA (7) has refueled the possibility of using specific characteristics of the RF and ACPA response as clinically useful biomarkers. In this short review, we will try to summarize the clinical associations of autoantibodies in RA also in light of their mechanistic contribution to disease processes.

## The pathogenic role of autoantibodies in RA

The past 10 years of research have shed increasing and exciting light on the multiple roles of autoantibodies (both RF and ACPA) in RA pathology, briefly summarized below.

### Pro-inflammatory role

**Figure 1:**
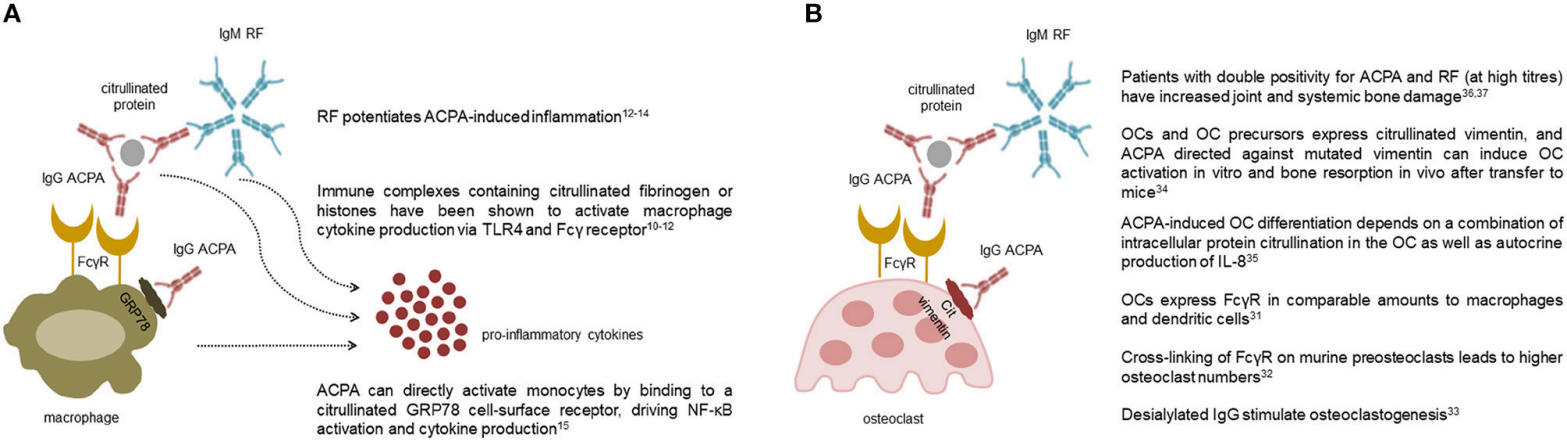
Pro-inflammatory and osteoclastogenic roles of autoantibodies in rheumatoid arthritis. The multiple effects of anti-citrullinated protein antibodies (ACPA) and rheumatoid factor (RF) on macrophages **(A)** and osteoclasts **(B)** are shown. Collectively, ACPA are able to stimulate cytokine production by macrophages both through the interaction of their Fc tail with stimulating Fcγ receptors, and through Fab-mediated recognition of membrane citrullinated proteins such as GRP78. Pentameric class M RF potentiates ACPA-induced inflammation likely via activation of the complement cascade **(A)**. ACPA are also able to promote osteoclast differentiation and activation through similar mechanisms mediated by their Fc and Fab portions. Again, clinical studies indicate that RF, especially at high titres, amplifies ACPA-mediate bone damage **(B)**.

All immunoglobulins (Ig), and in particular those of the IgG class, have effector functions mediated by the interaction of their Fc tail with specific activating or inhibitory receptors (FcR) present on the surface of various cell types. ACPA detected by second-generation assays are mostly of the IgG class, with predominance of the IgG1 subclass (8), which mediates Ig immunological activities (9). Through this mechanism, immune complexes containing ACPA are able to stimulate tumor necrosis factor (TNF) secretion by macrophages (10–12) (Figure 1A). This stimulus appears to be increased by the simultaneous presence of RF of the IgM and IgA classes, suggesting a synergism between ACPA and RF (12–14). In addition to FcR-mediated cell activation, ACPA can directly activate monocytes by binding through their Fab variable portion to a citrullinated GRP78 cell-surface receptor, driving NF-κB activation, and cytokine production (15) (Figure 1A). Another important effector function of Ig is represented by their ability to activate complement via both the classical and the alternative pathway. Complement activation is also demonstrated for ACPA (16), but is particularly effective in the case of the classic pentameric IgM RF (17). Accordingly, early studies have shown that aggregates of IgG-RF within the joints are associated with low complement levels and low C1q and C3 in synovial fluid (18). More recently, ACPA have been shown to influence inflammation and its chronicity also by increasing the generation of neutrophil extracellular traps (NETs) (19). NETs then stimulate joint inflammation by promoting the production of pro-inflammatory cytokines, chemokines, and adhesion molecules by synovial fibroblasts (19, 20).

Most of the pathological functions of autoantibodies in course of disease have been traditionally attributed to IgGs (macrophage activation through FcγR engagement) and IgMs (complement activation). However, recent evidence has also highlighted important effector functions of antibodies of the IgA class. Indeed, IgA potently activate neutrophils (21), and incubation of RA neutrophils with IgA immune complexes results in the release of NETs (22). These *in vitro* effects fit with clinical evidences linking the presence of IgA RF to worse disease prognosis (23, 24), and provide intriguing support to the possible mucosal origin of autoantibody responses in RA. Indeed, IgA represents the dominant Ig class in mucosal immune responses, and IgA plasmablasts dominate in ACPA-positive individuals at risk of developing RA (25).

Importantly, the unique effectors functions of autoantibodies are not only mediated by their diversification in classes and subclasses, but also by some specific biochemical characteristics including the sugar moiety attached to the Fc tail (26). In particular, antibodies carrying Fc glycans that lack galactose residues display an increased ability to activate the immune system (27, 28). Interestingly, a decrease in the level of galactosylation is one of the most prominent changes in total serum and ACPA IgG in course of RA (29, 30), whilst levels of anti-inflammatory glycans rise during drug-induced disease remission and pregnancy (31–33).

Altogether, the afore described mechanisms underline the complexity of the inflammatory function of autoantibodies in RA, which depends on intricate and not completely disentangled characteristics including classes, subclasses and biochemical properties. Better understanding of ACPA and RF diversity across the different phases of the disease will hopefully allow important advancements in the biological and clinical understanding of how inflammation develops, evolves and eventually resolves in RA.

### Osteoclastogenesis

The epidemiological association between ACPA and the severity of radiographic damage in RA has recently found causal explanation with the identification of the direct osteoclastogenic role of ACPA (34, 35). In general, immune complexes containing IgG, particularly those which have undergone modifications of their glycan residues, are able to bind to activating FcγR present on the surface of immature and mature osteoclasts and promote their activation (36–38) (Figure 1B). In addition, through their Fab portion, ACPA are able to bind to citrullinated vimentin present on the surface of osteoclasts and their monocyte-macrophage precursors (39), stimulating their differentiation and activation through interleukin (IL)-8 mediated circuits (40) (Figure 1B). Again, the process of ACPA-induced osteoclast activation seems to be amplified by the concomitant presence of RF, as indirectly suggested by radiographic studies which have shown increased amounts of joint and systemic bone loss in patients with double autoantibody-positivity, especially at high titres (41, 42). Altogether, such novel insights into the osteoclastogenic properties of ACPA have thus reinforced earlier concepts on the pathophysiological role of autoantibodies in RA by demonstrating their direct involvement in specific process of the disease such as bone damage.

### Nociception

Besides triggering inflammation and enhancing bone damage, a direct role of ACPA in promoting pain has also been suggested. Indeed, at least in experimental mouse models, the inoculation of ACPA triggers a pain reaction. Such mechanism seems to be mediated by the binding of ACPA to citrullinated epitopes on the surface of marrow osteoclast precursors, inducing the expression of CXCL1 (which is the murine analog of human IL-8), which in turn promotes nociceptive mechanisms (43). Although the nociceptive effect of ACPA *in vivo* remains to be fully proven, clinical evidences showing that ACPA-positive subjects may suffer from joint pain in the absence of overt synovitis (44) offer intriguing confirmation of this concept.

## Autoantibodies and clinical phenotype

It is now well-established that autoantibodies precede the onset of clinical symptoms by several years in a proportion of patients with RA (45, 46). The autoantibody response significantly evolves in the pre-clinical phase of the disease, with level increase, epitope spreading, isotype switching, affinity maturation, and change in glycosylation over time (47). Although the precise dynamics of such changes are not properly defined, and are likely to vary among different subjects, there is evidence that the extent of the autoantibody response conditions some clinical characteristics also in the very early phases of RA. Indeed, among autoantibody-positive subjects suffering from arthralgia, those at higher risk of quickly developing RA generally have higher autoantibody levels (48), more epitope spreading (49), and more frequently are ACPA and RF double positive (50). Thus, in a broader sense, autoantibody diversification may have an amplifying effect on inflammation and autoimmunity, leading to the transition from asymptomatic autoimmunity to complex responses triggering clinical inflammation. If this is the case, changes in autoantibody characteristics may serve as useful biomarkers to predict the development of RA and may represent valuable targets for preventive therapeutic strategies.

If early, pre-clinical autoimmunity remains an area of investigation, the clinical associations of autoantibodies in course of established RA have been abundantly analyzed. Literature dating back to the'80 was overall concordant in associating RF positivity, especially at high titers, to more inflammation (51, 52). As such, fluctuations in RF levels were proposed as a biomarker of disease activity and response to therapy (53). Also, RF of the IgA class was regarded as a specific diagnostic marker in early arthritis (54) and, in general, as a marker of more aggressive and refractory disease (23, 24). With ACPA capturing the scene from the 2000s, studies on early RA populations have massively focussed on this new autoantibody system, and interest on RF as a possible serological marker of inflammation has temporarily declined. In spite of their well-established association with more erosive disease, however, ACPA have soon appeared scarcely informative for patients' clinical phenotyping at presentation. In early RA patients from the Leiden early arthritis cohort, van der Helm-van Mil and co-authors (55) indeed reported similar symptoms and joint distribution between ACPA-positive and -negative patients. Number of swollen and tender joints, pain, functional status and acute phase reactants also appeared unaffected by the ACPA status in early RA patients from a Swedish cohort (56). Similarly, in 92 RA patients with symptoms of < 3 months' duration, the number of affected joints and inflammatory markers were comparable, and the only observed difference was the prevalence of knee involvement, slightly more common in ACPA-positive patients (57). More recent analyses have however relooked at clinical associations taking into account the interactions between RF and ACPA, rather than the two autoantibodies separately. In this setting, double autoantibody-positive patients seem to be characterized by a more inflammatory phenotype. In the French ESPOIR cohort, ACPA and RF double-positive patients presented with more marked elevation of acute phase reactants (58). Similarly, a large US study of 1,488 RA patients showed that double positivity for RF and ACPA was associated with significantly higher levels of C-reactive protein and pro-inflammatory cytokines (12). This was consistent with the amplifying effect of RF on TNF production triggered by ACPA *in vitro* (12). Analyzing the clinical data of patients enrolled in several clinical trials, Aletaha and collaborators (59) confirmed the boosting activity of RF on the background of ACPA, as ACPA single-positive patients had lower levels of disease activity. More recently, Derksen et al. (60) have nicely demonstrated in two large independent early RA cohorts that the amount of inflammation is proportional to the number of autoantibody-specificities, as patients with RF, ACPA and anti-carbamylated protein antibodies all positive had the highest levels of acute phase reactants. If these findings are confirmed, the simple distinction between autoantibody-positive and -negative patients would therefore appear insufficient at capturing different aspects of the disease. Rather, within autoantibody-positive patients, differences might exist depending on the diversity of the autoimmune response, as broader responses (indicating a break of tolerance to more autoantigens) would harbor more severe clinical phenotypes. It is however important to emphasize that the clinical association between the extent of the autoimmune response and disease activity described here does not allow to draw any conclusion on the cause-effect relationship between autoantibodies, in particular RF, and inflammation. On the one hand, the classic IgM RF could indeed represent a pro-inflammatory trigger, in light of its ability to activate complement (17, 18). On the other hand, RF production could be boosted by innate inflammatory stimuli outside a T cell-dependent germinal center reaction (61), therefore representing consequence rather than cause of inflammation.

Another aspect of the disease which appears to be strongly (and possibly mechanistically) linked to the presence of autoantibodies is extra-articular involvement, including cardiovascular (CV) comorbidity. Several studies have reported higher rates of CV mortality and events in autoantibody-positive patients (62, 63). This could be attributed to the higher inflammatory burden associated to autoantibodies, contributing to accelerated atherosclerosis, endothelial dysfunction, and plaque vulnerability (64). Supporting their direct pathogenic role, autoantibodies have been associated with CV events even in the absence of clinically apparent RA (65, 66). Indeed, irrespective of inflammation, autoantibodies might affect cardiac function, as suggested by studies indicating left ventricular strain, impaired left ventricular relaxation, and lower left ventricular mass in association with ACPA (67, 68). The direct effects of ACPA are in line with large epidemiological studies showing an increased rate of CV deaths specifically in ACPA-positive RA patients, whilst RF would be more associated to non-CV mortality (63).

It is however important to note here that the aforementioned associations between autoantibody diversity and local and systemic inflammation refer to patients fulfilling the 1987 classification criteria for RA. There is ample evidence now indicating that the introduction of the 2010 classification criteria has profoundly changed the clinical picture of RA (69). In this new setting, the high weigh attributed to autoantibodies in the scoring system has produced the paradoxical effect of making the clinical presentation of autoantibody-positive patients significantly milder compared to autoantibody-negatives (70, 71). Accordingly, ACPA-positive patients diagnosed with the 2010 criteria do not show more severe disease burden in terms of fatigue, pain, well-being, and independence compared to ACPA-negative patients (72). Also, with decreasing rates of incident CVD, the association between autoantibodies and comorbidities tends to disappear (73).

## Autoantibodies and disease remission

Disease remission is increasingly achievable in patients with RA provided that diagnosis is established early and treatment instituted effectively (74). However, the definition of remission is challenging, as it also incorporates the concepts of sustainability on treatment as well as persistence after drug suspension (75, 76). In this context, the predictive value of autoantibodies is difficult to establish. Furthermore, RF and ACPA could be related to slightly different remission outcomes.

In historical RA cohorts, RF has been shown to be inversely associated with the achievement of point remission in several studies. In the Utrecht RA cohort established in 1990, 36% of the patients achieved at least one period of remission; among other predictors, RF negativity was associated with 1.63 hazards of achieving remission (77). IgM RF positive patients were less likely to achieve remission also in other cohorts (78, 79), and a recent study on early RA patients commencing methotrexate (MTX) as their first disease modifying anti-rheumatic drug (DMARD) within 3 months of their baseline visit confirmed that RF was associated with earlier MTX failure due to inefficacy (80). In contrast, the predictive value of ACPA for achieving point remission is not that obvious. Surprisingly, studies in early RA cohorts diagnosed according to the 2010 classification criteria have shown that ACPA might rather be regarded as a marker of better outcomes, as ACPA-positive patients more frequently achieve remission upon induction therapy with high doses of MTX (81). Furthermore, broader autoantibody profiles in terms of specificities and classes associate with earlier response to treatment (82). Whether such findings also depend on the lower levels of disease activity in ACPA-positive patients diagnosed according to the 2010 criteria (70) remains to be demonstrated.

RF positivity not only appears associated with lower rates of point remission, but also with reduced chances of maintaining the state of disease remission over time. In a population of 650 RA patients followed for a mean of 10.3 years, Raheel et al. (83) were able to demonstrate that patients with positive RF had higher rates of flare compared to RF-negatives. In the IMPROVED study, positivity for RF was confirmed to be predictive of losing remission in course of drug tapering (84). Interestingly enough, in the same study, ACPA did not show a similar association, as ACPA-positive patients achieving early remission could taper medications similarly to ACPA-negatives (84). Very recently, data on the ARCTIC trial, in which treatment adjustments were tightly targeted on disease remission, have confirmed that RF, but not ACPA positivity was associated with not reaching sustained remission in univariate analysis (85).

If ACPA thus appear rather ininfluent in the achievement and maintenance of remission on treatment, their role in predicting disease relapse after drug suspension is undoubtedly established. In the Leiden Early Arthritis Clinic and the British Early Rheumatoid Arthritis Study, sustained DMARD-free remission was achieved far less commonly by ACPA-positive patients (86). Similar results were confirmed in the context of clinical trials (87), including again the IMPROVED study in which ACPA-positive patients showed more relapses in disease activity soon after drug suspension (84). This findings have solid evidence also in the recent RETRO trial (88–90). Here, ACPA-positivity and broader ACPA reactivities emerged as the strongest predictors of disease recurrence independent of the level of residual disease activity at drug tapering/discontinuation. Importantly, ACPA appear associated to increased hazards of losing remission irrespective of the type of drug being suspended, as studies on TNF and non-TNF drug tapering have shown similar results compared with conventional synthetic (cs) DMARD suspension (91).

Collectively, data from the literature thus indicate that both RF and ACPA are important factors related to remission outcomes. RF would mostly predict lower chances of achieving and maintaining remission on treatment, whilst ACPA would represent the major obstacle for safe drug suspension.

## Autoantibodies and response to therapy

### Response to conventional synthetic DMARDs

Despite being unanimously considered as important prognostic markers associated with more rapid evolution of joint damage, RA-specific autoantibodies, and in particular ACPA, have never been recommended as a guidance to assist the choice of first-line treatment in patients with early RA. Indeed, both the European and the American recommendations push for MTX as the anchor drug in all patients irrespective of autoantibody positivity (74, 92). This recommendation funds on evidences indicating that the worse radiographic progression observed in ACPA-positive RA is not a consequence of continuing disease activity, as response to therapy seems overall comparable irrespective of the serological subgroup. Among the several studies published so far, the more robust evidence of this concept probably comes from the long-term follow-up of the BEST strategy trial (93). Here, despite more radiological damage progression, ACPA-positive patients achieved reduction in disease activity similar to that of ACPA-negative patients in all treatment groups. Similarly, in the METEOR database including more than 1,800 patients with MTX as part of their initial treatment strategy, short-term remission in newly diagnosed RA was not affected by neither ACPA nor RF positivity (94). The analysis of ACPA reactivities does not seem to add much to prediction of treatment response, as recently shown in the context of the ARCTIC trial, were no difference in median baseline levels or number of ACPA reactivities was found between patients with different EULAR responses to MTX monotherapy (95).

If MTX efficacy appears overall similar between autoantibody-positive and –negative patients, still some differences in the magnitude of the response according to the serological status might exist. In patients with undifferentiated arthritis according to the 1987 criteria, MTX was effective at delaying progression to RA only in ACPA-positive patients, many of whom could be reclassified as having had RA according to the 2010 criteria at baseline (96). Similarly, in the IMPROVED study, ACPA-positivity was associated with higher chances of achieving remission upon induction treatment with MTX at the dose of 25 mg/week (81). In line with these data, results from our early arthritis inception cohort indicate that higher starting doses of MTX (15 mg/week) are more effective than lower doses (10 mg/week) in autoantibody-positive but not autoantibody-negative patients (97). The differential response to more aggressive strategies with csDMARDs also emerges from the CARDERA trial (98). Here, triple therapy with MTX, cyclosporine and prednisolone was more effective at reducing disease activity compared to MTX monotherapy only in ACPA-positive early RA patients.

Based on the above described evidences, it would be tempting to speculate that, by interfering with both the innate and the adaptive branches of the immune system (99), MTX at high doses is particularly effective in autoantibody-positive RA. However, one cannot exclude that the observed differences are actually related to misdiagnoses in patients lacking autoantibodies, rather than to intrinsic pathogenetic pathways differentially susceptible to different treatment strategies.

### Response to biological DMARDs

If the differential response to csDMARDs according to the autoantibody profile remains questionable, the impact of the serological status on response to biological (b) DMARDs targeting different immune pathways is, at least for certain mechanisms of actions, more defined.

It can be easily appreciated that bDMARDs selectively affecting the process of autoantibody production, including B cell depletion or inhibition of T cell costimulation, are particularly effective in autoantibody-positive patients. A meta-analysis of four randomized placebo-controlled trials from the rituximab clinical programme confirmed the additional treatment benefit in autoantibody-positive RA, particularly in patients for whom at least one TNF inhibitor had failed, and without significant differences between RF and ACPA (100). Similarly, a meta-analysis of 23 clinical trials and observational studies showed that positivity for RF at baseline predicted better response to rituximab according to both the ACR and the EULAR criteria (101). In recent times, ACPA positivity has emerged as particularly relevant in affecting the response to inhibition of T cell co-stimulation. A *post-hoc* analysis of the AMPLE trial in 2016 initially showed that very high levels of ACPA at baseline were associated with better clinical response to abatacept but not to adalimumamb (102). Such association has found confirmation in several independent real-life studies. In the French ORA registry, the proportion of ACPA-positivity was significantly higher among EULAR responders at 6 months (103). Data from other registries and real-world studies, such as the PanEuropean registry and the ACTION study, have shown that baseline autoantibodies impact on the retention rate of abatacept, as a surrogate marker of efficacy and safety (104, 105). Again, RF and ACPA appear both associated with better efficacy and lower risk of abatacept discontinuation for any reason (105, 106). It is also interesting to note that both B cell depleting agents and co-stimulation blockade selectively and equally affect not only RF, but also ACPA levels, thus confirming direct interference with adaptive immune responses (107). Although the clinical meaning of reduced ACPA titres upon treatment remains to be determined, initial evidence is starting to suggest that conversion to seronegative status may be associated with better clinical and radiographic outcomes (108).

Studies on the prognostic value of autoantibodies for response to therapy to bDMARDs targeting different mechanisms of action are less conclusive. The assessment of response to TNF inhibitors according to RF and/or ACPA status has provided conflicting results. A systematic literature review of 14 studies involving more than 5,000 patients treated with infliximab, etanercept, adalimumab, and golimumab demonstrated that neither RF nor ACPA were associated with differential response (109). However, the heterogeneity of the analysis was high, leaving results largely uncertain. Some studies have indeed highlighted lower rates of response in patients with high levels of RF (24, 110), particularly of the IgA class (24). These results have very recently been confirmed by *post-hoc* analyses on the RISING study, in which the dose of infliximab was progressively escalated (111, 112). In this study, patients with levels of RF and ACPA both high were characterized by higher levels of TNF, lower levels of infliximab and lower clinical response. Tociluzumab has even more discordant data. Indeed, response to IL-6 targeting does not seem to be affected by the ACPA status (113, 114). In contrast, high levels of RF would appear to favor tociluzimab treatment according to one report (114) and one meta-analysis (101). However, the mechanism of action of IL-6 antagonism in RA is complex and includes interference with both the innate and adaptive arms of the immune system. Accordingly, blockade of IL-6 receptor suppresses T- and B-cell activation signatures in RA synovium similarly to rituximab, but differently from TNF-antagonists (115). It is therefore very likely that those patients with the highest levels of activation of the B cell compartment also show preferential response to IL-6 targeting. In line with this concept, RA patients showing dense infiltration of lymphoid cells in their synovial membrane, as well as elevated serum levels of factors involved in B cell recirculation and positioning (such as the chemokine CXCL13), respond to tociluzumab better than to adalimumamb (116).

## Conclusions

**Figure 2:**
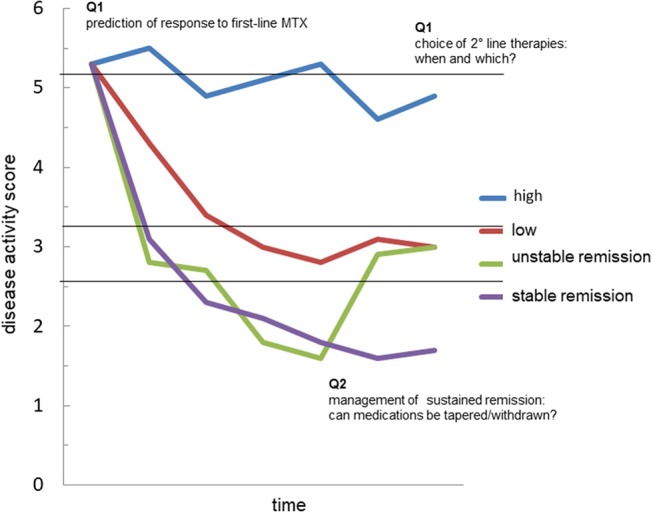
The possible value of autoantibodies as biomarkers in rheumatoid arthritis. The graph represents the possible trajectories of disease activity over time in patients with rheumatoid arthritis (RA): persistently high disease activity despite methotrexate (MTX) treatment (blue); satisfactory control of disease activity with a disease activity score indicative of low disease (red); achievement of remission, which is however unstable at follow-up (green); achievement of sustained remission (purple). Accurate prediction of such trajectories in every patient is currently an unmet need. The analysis of the fine characteristics of the autoantibody response in course of RA, including autoantibody levels, avidity, classes, subclasses and biochemical properties might generate useful biomarkers capable of answering critical questions such as: (Q1) the identification of those patients more likely to respond to first-line treatment with methotrexate (MTX); (Q2) the choice of second-line drug, among several mechanisms of actions available, in MTX-failures; (Q3) the possibility of tapering/stopping medications in patients achieving remission.

Given the complex pathophysiology and the clinical heterogeneity of RA, biomarkers capable of guiding personalized treatment approaches are urgently needed. RA would indeed benefit from personalized approaches across all the phases of disease development and evolution (Figure 2). Prompt recognition of at risk subject would indeed pave the way for preventive strategies. Soon after disease diagnosis, the precise identification of those patients in whom conventional, less expensive, and safe drugs such as MTX are more likely to be effective would allow significant savings for individuals and the society as a whole. Also, a more detailed and informed understanding of the phase of clinical remission would improve our ability to tailor treatment and follow-up to the individual patient. Several lines of evidence indeed indicate that, once remission has been achieved, the decision of tapering therapies is largely empirical, with high rates of insuccess. While awaiting new prognostic markers, rheumatologist may still obtain valuable information from traditional factors including autoantibodies. At present, neither RF nor ACPA can be reliably used as absolute markers of prognosis and response to treatment. However, the field of immunology is moving fast, and has opened new perspectives that, in the near future, could have important clinical translation. We believe that further refinement of the analytic procedures capable of capturing the broad spectrum of autoantibody characteristics, including classes, subclasses, specificities and biochemical properties, will rapidly allow better subclassification of patients with RA.

## Bullet points and research agenda

✓ The dichotomic classification of patients with RA into autoantibody-positive and -negative solely based on RF and/or ACPA appears too simplistic.
✓ The autoantibody system in RA is likely to expand rapidly with the identification of new antigenic specificities.
✓ The pathogenic properties of autoantibodies depend on several characteristics including levels, avidity, classes, subclasses, and biochemical properties.
✓ All of these properties are likely to be modulated in different patients during different phases of the disease.
✓ Further studies are needed to test whether the different characteristics of autoantibodies, alone or in combination, may serve as biomarkers for:
  - prediction of disease development in at risk individuals
  - prediction of response to MTX
  - choice of the second-line drug in MTX-failures
  - identification of patients in whom remission is more likely to persist even after drug tapering/discontinuation

## Author contributions

SB and AM prepared a first draft of the manuscript. RC and CM critically reviewed the manuscript. All authors conceived the idea of this review article and approved the final version.

### Conflict of interest statement

The authors declare that the research was conducted in the absence of any commercial or financial relationships that could be construed as a potential conflict of interest.
